# Gene signature and prediction model of the mitophagy-associated immune microenvironment in renal ischemia-reperfusion injury

**DOI:** 10.3389/fimmu.2023.1117297

**Published:** 2023-03-28

**Authors:** Ruo-Yang Chen, Da-Wei Li, Hui Xie, Xiao-Wen Liu, Shao-Yong Zhuang, Hao-Yu Wu, Jia-Jin Wu, Nan Sun, Jun-Wen Qu, Jia-Yi Miao, Chen Zhong, Yu-Hua Huang, Xiao-Dong Yuan, Ming Zhang, Wei-Jie Zhang, Jian-Quan Hou

**Affiliations:** ^1^ Department of Urology, The First Affiliated Hospital of Soochow University, Suzhou, China; ^2^ Department of Urology, Renji Hospital, Shanghai Jiaotong University, School of Medicine, Shanghai, China; ^3^ Department of Institute of Molecular Medicine, Renji Hospital, Shanghai Jiaotong University, School of Medicine, Shanghai, China; ^4^ Department of Urology, Dushu Lake Hospital Affiliated to Soochow University, Suzhou, China

**Keywords:** gene signature, mitophagy, immune microenvironment, prediction model, renal ischemia-reperfusion injury

## Abstract

**Background:**

Renal ischemia-reperfusion injury (IRI) is an inevitable occurrence during kidney transplantation. Mitophagy, ferroptosis, and the associated immune microenvironment (IME) have been shown to play important roles in renal IRI. However, the role of mitophagy-associated IME genes in IRI remains unclear. In this study, we aimed to construct a prediction model of IRI prognosis based on mitophagy-associated IME genes.

**Method:**

The specific biological characteristics of the mitophagy-associated IME gene signature were comprehensively analyzed using public databases such as GEO, Pathway Unification, and FerrDb. Correlations between the expression of prognostic genes and immune-related genes and IRI prognosis were determined by Cox regression, LASSO analysis, and Pearson’s correlation. Molecular validation was performed using human kidney 2 (HK2) cells and culture supernatant as well as the serum and kidney tissues of mice after renal IRI. Gene expression was measured by PCR, and inflammatory cell infiltration was examined by ELISA and mass cytometry. Renal tissue damage was characterized using renal tissue homogenate and tissue sections.

**Results:**

The expression of the mitophagy-associated IME gene signature was significantly correlated with IRI prognosis. Excessive mitophagy and extensive immune infiltration were the primary factors affecting IRI. In particular, FUNDC1, SQSTM1, UBB, UBC, KLF2, CDKN1A, and GDF15 were the key influencing factors. In addition, B cells, neutrophils, T cells, and M1 macrophages were the key immune cells present in the IME after IRI. A prediction model for IRI prognosis was constructed based on the key factors associated with the mitophagy IME. Validation experiments in cells and mice indicated that the prediction model was reliable and applicable.

**Conclusion:**

We clarified the relationship between the mitophagy-related IME and IRI. The IRI prognostic prediction model based on the mitophagy-associated IME gene signature provides novel insights on the prognosis and treatment of renal IRI.

## 1 Introduction

Ischemia-reperfusion injury (IRI) is defined as the paradoxical deterioration of cellular dysfunction and death after blood flow is restored to previously ischemic tissue (1, 2). The reconstruction of blood flow is essential to salvage ischemic tissue. However, it is a double-edged sword, as reperfusion can cause further damage and threaten normal organ function and viability (3, 4).

Renal tissues are sensitive to IRI (5), and IRI is an inevitable challenge in renal transplantation under clinical settings. A thorough exploration of the molecular and cellular mechanisms is essential to understand the possible windows for effective therapeutic intervention. To date, many studies have shown the primary mechanisms leading to renal IRI. These include impaired energy metabolism, calcium overload, mitochondrial damage, oxidative stress response, inflammatory response, apoptosis, and autophagy (6, 7).

Renal tubular epithelial cells are the cells most severely damaged in response to IRI (8). IRI results in the loss of tubular epithelial cell function, leading to acute renal injury, delayed graft function, and acute and chronic organ rejection (9, 10). Mitochondrial autophagy and ferroptosis also occur in renal tubules in response to IRI (11, 12).

Among these factors, mitochondrial autophagy acts as a trigger for the process throughout IRI and interacts sequentially with other forms of cell death, most often ferroptosis (13). Mitochondrial autophagy is a key cellular homeostatic mechanism that is activated early in IRI through PRKN-dependent and -independent signaling pathways (14). In this regard, the activation of mitochondrial autophagy exerts a protective effect, reducing local inflammation and oxidative damage. Instead, the crosstalk between mitochondrial autophagy and forms of cell death induces direct tissue damage (15, 16). Findings from many studies have suggested an important relationship between mitochondrial autophagy and ferroptosis (17, 18).

However, the transcriptomic differences in the development in renal IRI and the degree of interaction and impact on mitochondrial autophagy and ferroptosis are yet to be explored (19, 20). To this end, our study first identified key differentially expressed genes through transcriptome analysis. Subsequently, we analyzed the association between typing and prognosis and observed a difference in the survival time. Finally, we verified the occurrence of mitochondrial autophagy and ferroptosis after renal IRI in cellular and mouse models, which eventually led to a significant increase in inflammatory infiltration.

In this study, we explored the mechanisms underlying renal tubular injury and the process of injury development at the molecular level in human samples and validated them in cellular and mouse models. Our findings would be helpful in therapeutic applications for renal IRI.

## 2 Materials and methods

### 2.1 Data acquisition and pre-processing

The microarray datasets and corresponding annotation files of transcriptomic datasets with reliable renal IRI specimens [GSE43974 (21), GSE90861 (22), GSE126805 (23), and GSE21374 (24)] were retrieved from the Gene Expression Omnibus (GEO). The GSE43974 dataset, which included 203 renal IRI specimens and 188 control specimens collected before IRI from brain-dead donors (DBD), cardiac dead donors (DCD), and living donors (LD), was used for analyzing differentially expressed mitophagy/ferroptosis-related genes. The GSE90861 dataset includes 23 renal IRI specimens and 23 pre-IRI control specimens. The GSE126805 dataset includes 42 renal IRI specimens and 41 pre-IRI control specimens. These two datasets were used as the validation dataset for differentially expressed genes (DEGs). GSE21374 contains the transcriptomic and clinical prognosis data of 282 renal IRI specimens and was used for identifying prognostic genes and risk scoring (**Figure S1**).

### 2.2 Differential expression of mitophagy/ferroptosis-related genes and validation

A list of mitophagy-related genes (MRGs) was acquired from the Pathway Unification database, and 26 MRGs were extracted from the transcriptomic data of GSE43974 (**Table S1**). A list of ferroptosis-related genes (FRGs) was acquired from FerrDb (Methods, **Supplementary Material**), and 248 FRGs were extracted from GSE43974 (**Table S2**). Differentially expressed MRGs/FRGs between the renal IRI samples and control samples in GSE43974 were identified using the results of the Wilcoxon test. A heatmap of differentially expressed MRGs/FRGs was constructed using the pheatmap package. Correlations between MRG and FRG expression were determined using Pearson’s correlation analysis. The differentially expressed MRGs/FRGs were subsequently validated using GSE90861 and GSE126805.

### 2.3 The protein-protein interactionnetwork and transcription factor, miRNA, and small molecule compound networks

The PPI network of the differentially expressed MRGs/FRGs was constructed using Search Tool for the Retrieval of Interacting Genes (25) and Cytoscape (26). The transcription factor (TF), miRNA, and small molecule compound networks of the differentially expressed MRGs/FRGs were also analyzed.

### 2.4 Molecular subtyping of renal IRI

Molecular subtyping was performed using the non-negative matrix factorization (NMF) package (27) based on gene expression in the GSE21374 dataset (top 5000 genes in decreasing variance). According to the change in the cophenetic coefficient with K-means, the optimal number of clusters was determined by the rank before the point of maximum change. Differentially expressed MRGs/FRGs and differences in prognosis were analyzed between subtypes.

### 2.5 Prognostic marker selection

Prognostic markers were selected from the GSE21374 dataset using LASSO regression. Variables were selected by the glmnet function and cross-validated by the cv.glmnet function in the glmnet package (28) to obtain the combination of prognostic markers with the minimum CV coefficient.

### 2.6 Risk score and risk grouping

The optimal risk score (RS) cutoff for predicting survival time in kidney transplant recipients was determined using the maxstat package. Patients were divided into the low RS and high RS groups using the cutoff, and survival curves were plotted using the Kaplan-Meier (K-M) estimator. The ability of RS to predict the 1-, 2-, and 3-year survival was analyzed using the survival receiver operator characteristic curve (ROC) package, and the area under a curve (AUC) of the ROC curve was calculated.

### 2.7 Differential gene expression and functional enrichment analyses of risk groups

DEGs between the two risk groups were analyzed to elucidate the biological significance of RS. DEGs were identified using an adjusted P value < 0.05 and |log2FC| >1. The volcano plot and heatmap of DEGs were generated using ggplot2 and pheatmap, respectively. Kyoto Encyclopedia of Genes and Genomes (KEGG) and Gene Ontology (GO) analyses were performed on the DEGs of each risk group using clusterProfiler.

### 2.8 Immune cell infiltration analysis

The gene expression matrix of GSE21374 was analyzed using CIBERSORTx to determine immune cell infiltration in the samples. Samples with significant infiltration were identified by a *P* < 0.05. The extent of immune cell infiltration was compared between the low and high RS groups as well as between molecular subtypes using the Wilcoxon test. The correlation between immune cells and the candidate MRGs/FRGs was determined by Pearson’s correlation analysis.

### 2.9 Construction of a cellular model of IRI

HK2 cells were subjected to hypoxia and reoxygenation to confirm mitochondrial damage, mitophagy, and ferroptosis in response to hypoxia-reoxygenation injury (HK2 cells were purchased from the Cell Bank of Type Culture Collection of Chinese Academy of Sciences and cryopreserved in an ultra-low temperature freezer in our laboratory). HK2 cells at an appropriate density were cultured in DMEM-F12 (Gibco-12634010) supplemented with 10% premium-grade fetal bovine serum (Gibco-16000-044) at 37 °C with 5% CO_2_ for 12-24 h until adherence was observed. The cells were then divided into the normoxia group (n = 3) and hypoxia group (n = 3) according to the experimental design.

HK2 cells in the normoxia group were cultured at 37 °C with 5% CO_2_, while those in the hypoxia group were cultured under hypoxic conditions. Briefly, the inlet and outlet valves of the hypoxia chamber were opened, and a mixed gas of 94% N_2_/5% CO_2_/1% O_2_ was injected into the chamber at a flow rate of 2L/min for 5 min. Once the oxygen level was stabilized at 1%, the outlet valve was closed and the cells were cultured for 24 h. The cells were then reoxygenated in a conventional incubator (5% CO_2_, 21% O_2_, 74% N_2_) for 4 h.

HK2 cells were collected from both groups, and the level of inflammatory cytokines in the culture supernatant was measured using an ELISA KIT (Human IL-6 ELISA KIT, Abcam, ab100572, Human TNFa ELISA KIT, Abcam, ab285312, Human CXCL1 ELISA KIT, Abcam, ab190805). Cell necrosis and cell apoptosis were assessed using Annexin V-FITC flow cytometry (Beyotime-C1062). Changes in the mitochondrial membrane potential were examined by fluorescence microscopy (enhanced mitochondrial membrane potential assay kit with JC-1, Beyotime-C2003S). The expression of ferroptosis markers (MDA, GSH, GPX4, and NADPH) was detected by biochemical testing (Nanjing Jiancheng, MDA kit, GSH-PX kit, GPX4 Kit, and NADPH kit).

### 2.10 Animal model of IRI

SPF wild-type (WT) C57BL/6 mice (6-8 weeks old, 23.0-25.0 g) were purchased from the Shanghai Laboratory Animal Center and acclimatized in our animal room for at least 1 week. Animals were housed at 24 ± 1°C under 40% ± 1% relative humidity and a 12 h/12 h light/dark cycle, with no more than five animals per cage, and were provided ad libitum access to food and water. All experimental procedures were reviewed and approved by the animal ethics committee. The mice were divided into the sham group (excision of right kidney only) and IR model group (excision of right kidney and 25 min of left renal artery clamping followed by 24 h of blood reperfusion),and six in each group.

Blood and kidney tissues were collected from the mice at 24 h after surgery. The serum creatinine level was measured using an automated biochemistry analyzer, and the IL-6, TNF-α, CXCL1 were measured by ELISA KIT (Mouse IL-6 ELISA KIT, Solarbio, SEKM-007, Mouse TNFa ELISA KIT, Solarbio, SEKM-0034, Mouse CXCL1 ELISA KIT, Solarbio, SEKM-0046), and ferroptosis marker levels were also measured. Gene expression in the kidney tissues was determined by PCR (the primer sequences are shown in **Supplementary Table S5**). HE and TUNEL staining was performed on the remaining kidney tissues for histopathology and evaluation of the extent of IRI, respectively. In addition, mitochondrial damage and autophagosomes in the kidney tissues were examined using transmission electron microscopy (TEM). The renal immune microenvironment was evaluated by single-cell mass spectrometry-flow cytometry.

### 2.11 Statistical analysis

Data calculation and statistical analyses were performed by R (https://www.r-project.org/, version 4.2.0). The false positive rate of multiple testing was reduced using Benjamini-Hochberg multiple testing correction. Continuous variables with normal distribution were compared using the independent *t*-test, and variables with non-normal distribution were compared using the Mann-Whitney U test (e.g., Wilcoxon rank-sum test). ROC curves were generated using the survival ROC, and the area under the ROC curve (AUC) was calculated to evaluate the accuracy of RS in predicting prognosis. Survival was analyzed by Cox regression, and the hazards ratio (HR), 95% confidence interval (CI), and RS cutoff were calculated. All tests were two-tailed, and a *P* < 0.05 was considered statistically significant.

## 3 Results

### 3.1 MRG/FRG expression

**Figure 1:**
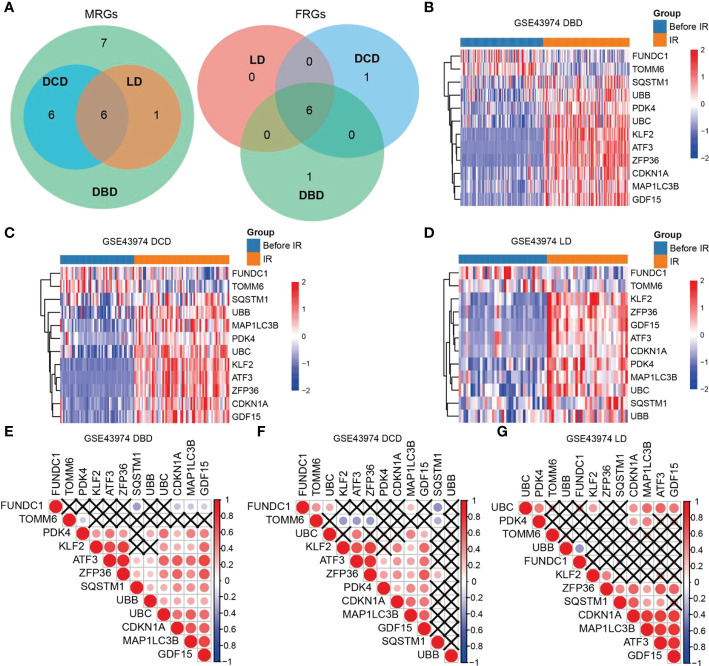
Heatmaps and correlation analysis of differentially expressed MRGs/FRGs. **(A)** Venn diagram of differentially expressed MRGs/FRGs in DBD, DCD and LD. All 6 MRGs and 6 FRGs were differentially expressed in the 3 subject populations. **(B–D)** Heatmaps of MRGs/FRGs expression in samples from **(B)** DBD, **(C)** DCD, and **(D)** LD. **(E–G)** Correlation matrices of MRGs/FRGs expression in DBD, DCD and LD. Red indicates positive correlation and blue indicates negative correlation. A darker indicates a higher correlation (Pearson coefficient), and a black X indicates no statistical significance. MRGs, mitophagy-related genes; FRGs, ferroptosis-related genes; DBD, brain-dead donor; DCD, cardiac dead donor; LD, living donor; IR, ischemia reperfusion.

We compared the expression of MRGs/FRGs between the IR and pre-IR samples of DBD, DCD, and LD and identified six MRGs and six FRGs that were significantly differentially expressed in the three groups (**Figure 1A**). MRG/FRG expression in the different samples is shown in heatmaps (**Figures 1B–D**). The differentially expressed MRGs included FUN14 domain containing 1 (FUNDC1), translocase of outer mitochondrial membrane 6 (TOMM6) (both downregulated), microtubule-associated protein 1-light chain 3B (MAP1LC3B), sequestosome 1 (SQSTM1), ubiquitin B (UBB), and ubiquitin C (UBC) (all upregulated). The differentially expressed FRGs included activating transcription factor 3 (ATF3), Kruppel 2 (KLF2), zinc finger protein 36 (ZFP36), cyclin-dependent kinase inhibitor 1A (CDKN1A), growth differentiation factor 15 (GDF15), and pyruvate dehydrogenase kinase (PDK4) (all upregulated). The correlation of MRG and FRG expression was also detected by correlation analysis. The correlation matrices are shown in **Figures 1E–G**. Next, we validated the 12 differentially expressed MRGs/FRGs using the GSE90861 and GSE126805 datasets and found that all 12 MRGs/FRGs were differentially expressed in the two datasets, with trends of expression consistent with those identified in our analysis (**Figure S2**).

### 3.2 PPI, miRNA, TF, and small molecule compound interaction networks of differentially expressed MRGs/FRGs

We constructed a PPI network of the 12 differentially expressed MRGs/FRGs (**Figure S3A**) and found that ZFP36, UBC, GDF15, MAP1LC3B, CDKN1A, SQSTM1, TOMM6, UBB, ATF3, KLF2, PDK4, and FUNC1 were associated with 187, 186, 119, 116, 104, 75, 59, 59, 43, 12, 12, and 2 (ZNF71 and MAZ) transcription factors (TFs), respectively (**Figure S3B**). The miRNA interaction analysis revealed that CDKN1A, KLF2, ZFP36, UBC, UBB, MAP1LC3B, SQSTM1, ATF3, GDF15, and PDK4 were associated with 331, 107, 53, 49, 42, 42, 27, 24, 23, and 6 (hsa-mir-16-5p, hsa-mir-26b-5p, hsa-mir-103a-3p, hsa-mir-182-5p, hsa-mir-122-5p, and hsa-mir-335-5p) miRNAs, respectively (**Figure S3C**). Furthermore, the small molecule compound interaction analysis showed that CDKN1A, ATF3, GDF15, SQSTM1, MAP1LC3B, ZFP36, PDK4, KLF2, UBC, UBB, FUNDC1, and TOMM6 were associated with 618, 204, 202, 166, 95, 72, 56, 45, 29, 22, 6 (acetaminophen, catechin, cyclosporine, grape seed proanthocyanidins, K7174, and tetrachlorodibenzodioxin), and 7 (aflatoxin B1, arsenic, chloropicrin, copper sulfate, cyclosporine, K7174, and valproic acid) small molecule compounds, respectively (**Figure S3D**).

### 3.3 Molecular subtyping

**Figure 2:**
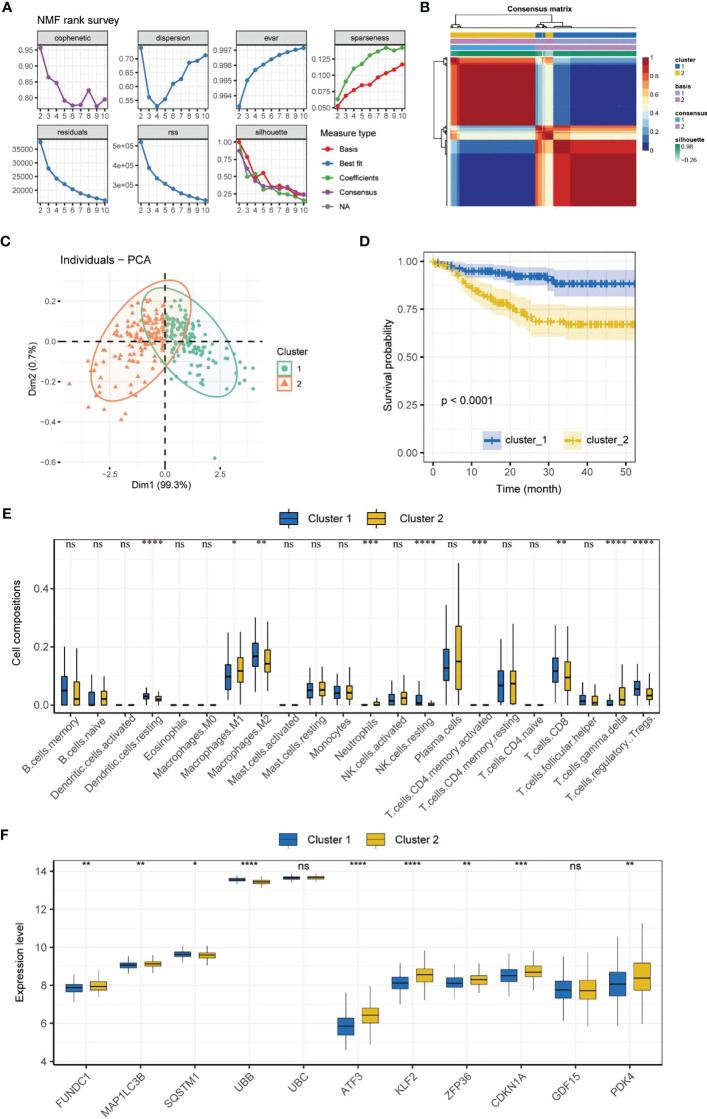
Molecular subtyping. **(A)** Process of NMF subtype clustering. **(B)** Heatmap of subtypes. **(C)** PCA of subtypes. **(D)** Survival curves of subtypes. (K–M method). **(E)** Box plot of immune cell infiltration in each subtype. **(F)** Box plot of MRGs/FRGs expression in each subtype. Gene expression in the box plot was compared using the Wilcoxon test. ns, no significance, *P < 0.05, **P < 0.01. ***P < 0.001, ****<0.0001. NMF, non-negative matrix factorization; PCA, principal component analysis.

NMF subtyping showed that the cophenetic coefficient began to decrease at rank=2, which indicated that the optimal number of subtypes was 2 (**Figure 2A**). A heatmap of the subtypes is shown in **Figure 2B**. Principal component analysis showed that the samples could be clearly distinguished by the two subtypes (**Figure 2C**). Correlation analysis of the subtype and prognosis revealed that the survival time differed significantly between subtypes, and subtype 2 patients had better prognosis than subtype 1 patients (**Figure 2D**). The analysis of immune cell infiltration showed that subtype 1 patients had significantly higher infiltration of resting dendritic cells, M2 macrophages, resting NK cells, CD8+ T cells, and regulatory T cells (Tregs) (P < 0.01), whereas subtype 2 patients had significantly higher infiltration of M1 macrophages, neutrophils, activated memory CD4+ T cells, and gamma delta T cells (P < 0.05) (**Figure 2E**). The expression of MRGs/FRGs, namely FUNDC1, MAP1LC3B, SQSTM1, UBB, ATF3, KLF2, ZFP36, CDKN1A, and PDK4, was significantly different between the two subtypes (*P* < 0.05) (**Figure 2F**).

### 3.4 Prognostic marker selection and risk score calculation and evaluation

We selected seven MRGs/FRGs (FUNDC1, SQSTM1, UBB, UBC, KLF2, CDKN1A, and GDF15) as prognostic markers using LASSO regression (**Figures S4A, B**). The AUC of the ROC curve was 0.73, indicating good predictive ability (**Figure S4C**). The survival curves of the seven candidate genes in the high/low expression groups are shown in **Figures S4 D-J**. The correlation analysis of candidate gene expression and immune cell infiltration is shown in **Figure S4K**. The risk score (RS) was calculated based on the coefficients of the candidate prognostic markers determined by LASSO regression (Methods are shown in the **Supplementary Material**).

RS=0.3038*FUNDC1+(-0.5346)*SQSTM1+(-3.5716)*UBB+2.3143*UBC+0.4939*KLF2+0.2815*CDKN1A+0.0487*GDF15.

**Figure 3:**
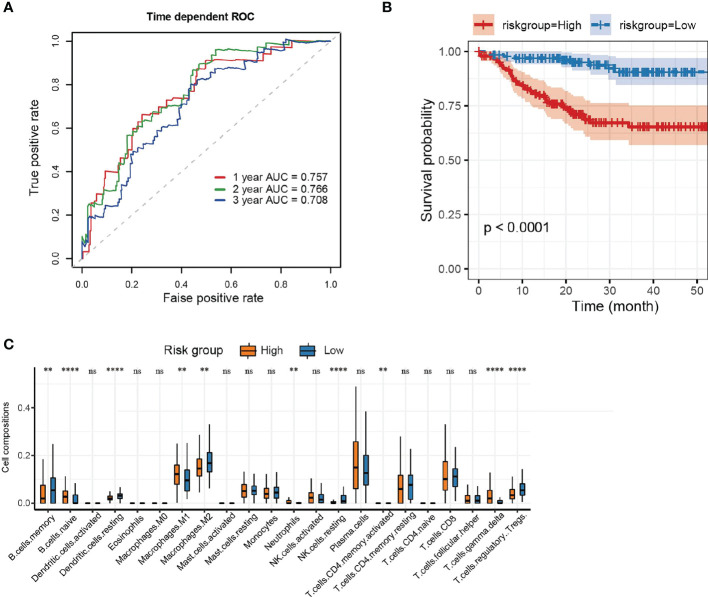
Risk score and evaluation. **(A)** ROC curves of RS in predicting the 1-, 2- and 3-year survival of kidney transplant patients. **(B)** Survival curves (K–M method) of the low and high RS groups. **(C)** Box plot of immune cell infiltration in the low and high RS groups. Data were compared using the Wilcoxon test. **P <0.01, ****P < 0.0001, ns, no significance.

The ROC curves of RS for predicting survival showed good predictive ability (**Figure 3A**). Using maxstat, we determined that the best RS cutoff for predicting the survival of patients who underwent kidney transplant was -25.0716. We then categorized patients who underwent renal transplant into the high RS and low RS groups based on this cutoff and removed patients without survival information. Prognosis was significantly poorer in patients with a high RS than in patients with a low RS (*P* < 0.0001) (**Figure 3B**).

In addition, we compared the extent of immune cell infiltration between the high RS and low RS groups (**Figure 3C**). Our analysis revealed that the high RS group had a greater infiltration of B cells, neutrophils, T cells, and M1 macrophages (P < 0.01), whereas the low RS group had a greater infiltration of memory B cells, dendritic cells (DCs), M2 macrophages, resting NK cells, regulatory T cells (Tregs), and T cells (P < 0.01).

### 3.5 Differential gene expression and enrichment analyses in RS groups

We performed enrichment analysis of these DEGs (**Tables S3**, **S4**). KEGG analysis showed that the DEGs were enriched in *Staphylococcus aureus* infection, viral protein interaction with cytokine and cytokine receptor, chemokine signaling pathway, protein digestion and absorption, and pertussis (**Figures S5A-D**).

Furthermore, GO analysis revealed that the DEGs were associated with BPs such as humoral immune response, leukocyte mediated immunity, adaptive immune response based on somatic recombination of immune receptors built from immunoglobulin superfamily domains, lymphocyte mediated immunity, and activation of immune response, MFs such as antigen binding, CXCR chemokine receptor binding, and glycosaminoglycan binding and heparin binding, and CCs such as external side of plasma membrane, blood microparticle, collagen-containing extracellular matrix, collagen trimer, and secretory granule lumen (**Figures S5E, F**).

### 3.6 Hypoxia results in HK2 cell injury

**Figure 4:**
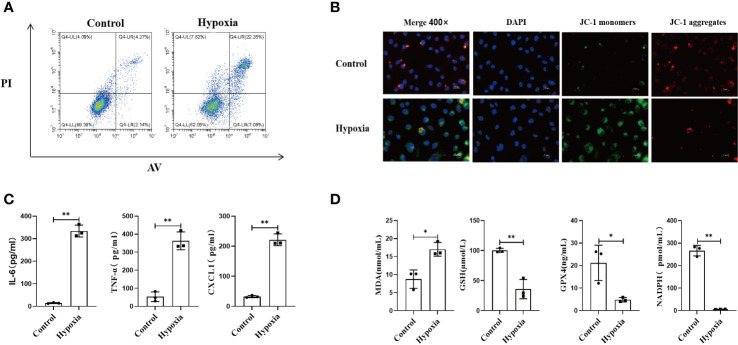
HK2 cells under hypoxic environment would cause the occurrence of ferroptosis, and accompanied by the secretion of inflammatory factors. **(A)** HK2 cells were cultured in hypoxia for 24 h and reoxygenated for 4 h, and the apoptotic and necrotic ratios were determined using flow cytometry, **(B)** Mitochondrial membrane potential alteration was detected by JC-1 probes, Scale bar, 50 um and 25 um. **(C)** Inflammatory factors secreted by HK2 within cell culture supernatants were examined, **(D)** Cell culture supernatant were assayed for markers of ferroptosis. n = 3, *P < 0.05, **P < 0.01 between groups as indicated.

Cell viability in the normoxia and hypoxia groups was measured by flow cytometry. Our results demonstrated that the numbers of necrotic and apoptotic HK2 cells were far higher in the hypoxia group than in the normoxia group (**Figure 4A**), along with increased intracellular ROS level (**Figure S6**). We also found that the mitochondrial membrane was depolarized after hypoxia, which suggested that hypoxia could impair mitochondrial functions (**Figure 4B**).

Next, we examined the inflammatory response of HK2 cells under different conditions by collecting the culture supernatants. Hypoxia upregulated the expression of inflammatory mediators in HK2 cells (**Figure 4C**). Furthermore, the expression of ferroptosis-related markers was also increased in the HK2 cell culture supernatant after hypoxia, which indicated that hypoxia exacerbated the ferroptosis of HK2 cells (**Figure 4D**). Collectively, these findings indicate that hypoxia leads to renal tubular epithelial cell injury along with increasing inflammatory mediator expression, mitophagy, and ferroptosis.

### 3.7 IRI led to the impairment of renal function in mice

**Figure 5:**
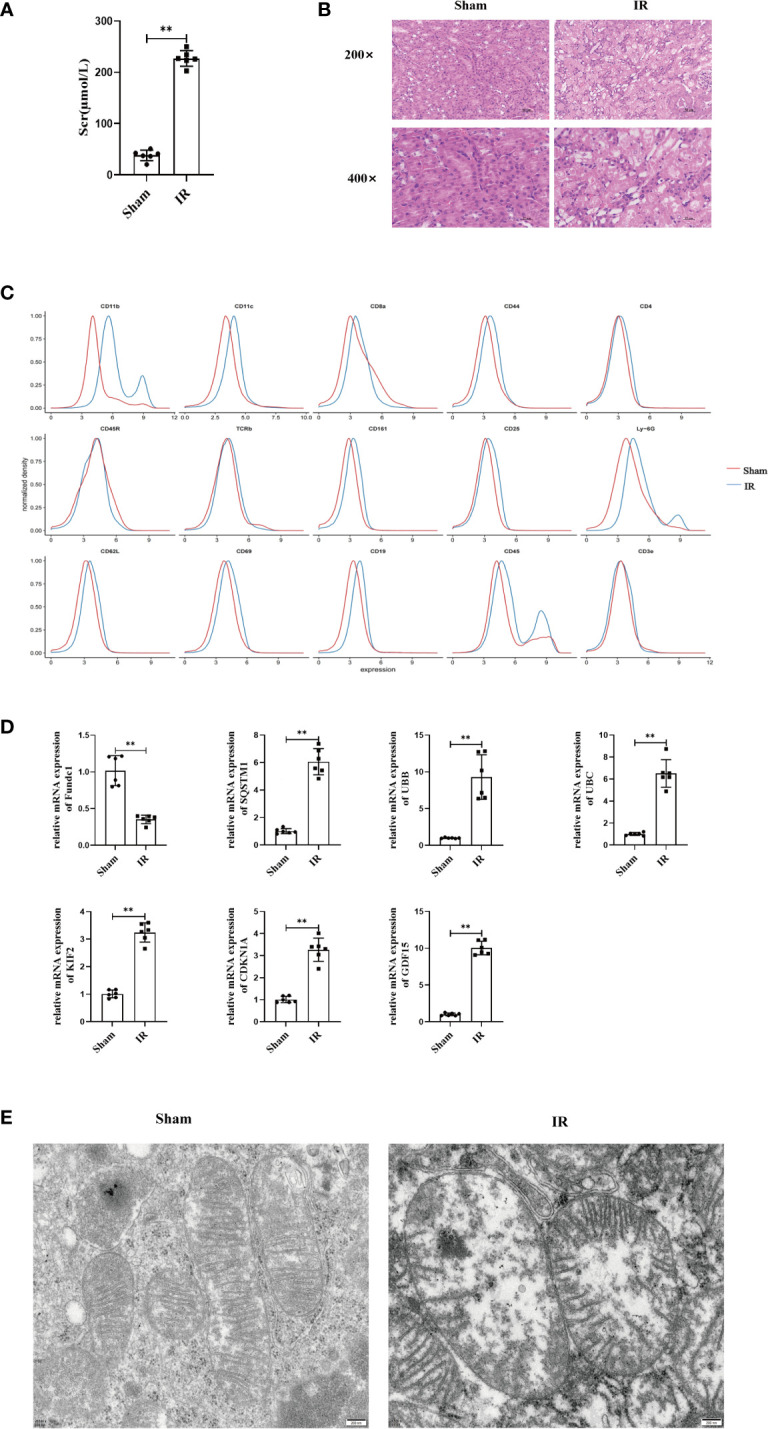
Renal IRI causes renal insufficiency and in mice. **(A)** Secrum creatinine was detected in different groups of mice and found that IR was higher than Sham group, **(B)** HE staining of mouse kidney tissue, can see that IR can lead to shedding necrosis and vacuole-like degeneration of tubular epithelial cells, Scale bar, 50 um and 25um. **(C)** Using single cell flow technology, **(D)** PCR to determine the expression of kidney tissue expression of MRGs and FRGs, **(E)** Electron microscopic photography showed significant changes in mitochondrial structure after IR. Scale bar, 200 nm. n = 6, **P < 0.01 between groups as indicated.

We compared the renal functions of mice in the sham and IR groups and found that renal IRI led to the impairment of renal function (**Figure 5A**). IRI resulted in severe renal tubular damages, as indicated by renal tubular epithelial cell necrosis and sloughing (**Figure 5B**). TUNEL staining results also indicated that IRI caused severe renal damage (**Figure S7**). Next, we found that inflammatory mediators were significantly overexpressed in the model group compared to that in the sham group (**Figure S8**). Furthermore, mass spectrometry-flow cytometry revealed that renal IRI significantly increased immune cell infiltration into the kidneys (**Figure 5C**). The levels of ferroptosis markers differed significantly in the model group compared to that in the sham group (*P* < 0.05) (**Figure S9**). In addition, we also observed the expression of FRGs and MRGs in the kidney tissues, indicating that IRI could upregulate FRG and MRG expression induced mitophagy (**Figure 5D**). TEM confirmed that renal IRI led to mitochondrial atrophy, with a reduction in the disappearance of mitochondrial cristae and increase in mitochondrial membrane density and autophagosome abundance (**Figure 5E**).

## 4 Discussion

The clinical management of IRI has been highly challenging. Although many approaches validated in animal models have shown considerable promise in reducing the severity of IRI, the results of clinical trials have not been satisfactory (29, 30).

Owing to the special tissue structure and function of the kidneys, they are extremely sensitive to IRI (31). In renal transplantation, the graft inevitably undergoes a series of changes, such as donor hemodynamic disturbance, thermal ischemia, cold ischemia, and reperfusion, which causes IRI, leading to delayed recovery in renal function post-transplantation, primary non-function of transplanted kidneys, rejection, or even the loss of transplanted kidneys (32). These remain important factors affecting the early recovery of renal function and long-term survival of the graft (33). Thus, the prevention and treatment of IRI in transplanted kidneys have become an important topic for research in the field of renal transplantation in recent years.

The regulation of mitochondrial kinetics, which manifests as a dynamic balance between the processes of mitochondrial fusion and division, is closely associated with the generation of reactive oxygen species (ROS) and Ca^2+^ in large quantities and high energy metabolism during renal IRI. It is an important factor affecting cell survival or death. Imbalances in mitochondrial kinetics can lead to the disturbance of the intracellular environment, cell damage, and even death (34–36), thus leading to renal tubular necrosis (37). Changes in mitochondrial function can also promote the increase in ROS content, thus forming a vicious circle.

More interestingly, mitochondrial autophagy also interacts with ferroptosis, because ferroptosis is an iron-dependent lipid peroxidation-mediated mechanism of cell death, and the mitochondria, as intracellular “oxidation factories” and effectors of oxidative stress, are inextricably linked to ferroptosis (18, 38, 39).

Consistent with this, our study showed that the ferroptosis-related genes ATF3, KLF2, ZFP36, CDKN1A, GDF15, and PDK4 are upregulated in renal IRI. This is a direct cause of renal injury, which suggests the need for the early prediction of renal IRI and intervention. Meanwhile, we also observed the expression of mitophagy-related genes in IRI.

In this study, the combined prediction of the ROC curve of FUNDC1, SQSTM1, UBB, UBC, KLF2, CDKN1A, and GDF15 was confirmed by the LASSO regression results. The results confirmed a relatively good prognostic predictive ability and provided a basis for the early diagnosis of renal IRI at the molecular level.

Notably, the findings of this study not only provide evidence at the molecular level but also validated the association of renal IRI with mitochondrial autophagy and ferroptosis at the cellular and animal-model levels. In the HK2 cell model, we confirmed that hypoxia can cause damage to renal tubular epithelial cells and is accompanied by the high expression of inflammatory factors and the occurrence of mitochondrial autophagy and ferroptosis. In a mouse renal IRI model, we confirmed the significant elevation of the indicators of ferroptosis in the kidneys, which was accompanied by mitochondrial atrophy.

In summary, the mitochondria are highly susceptible to damage under stressful conditions such as IRI, which leads to functional disorders. The kinetic regulatory mechanisms between mitochondrial division and fusion were demonstrated in clinical human samples, HK2 cells, and mouse models of acute renal IRI. Maintaining homeostasis in mitochondrial dynamics could be a novel research direction for mitigating acute IRI and may be a new target for drug action. However, further investigation and validation are needed for clinical implementation.

## 5 Conclusions

The findings of this study confirmed that mitochondrial autophagy and ferroptosis are closely related to the prognosis of renal IRI and possess important theoretical and practical implications for investigations on renal protection protocols during renal transplantation.

## Data availability statement

The original contributions presented in the study are included in the article/**Supplementary Material**. Further inquiries can be directed to the corresponding authors.

## Ethics statement

The animal study was reviewed and approved by the ethics committee [20220704(12)].

## Author contributions

R-YC, D-WL: Data curation, writing-original draft preparation. HX, X-WL: Bioinformatics and the analysis, S-YZ, H-YW, J-JW, NS: Construction of a cellular model of IRI, J-WQ, J-YM, CZ: Animal model of IRI, Y-HH, X-DY, MZ, W-JZ, J-QH: Writing-reviewing and editing. All authors critically revised the manuscript, agree to be fully accountable for ensuring the integrity and accuracy of the work. All authors contributed to the article and approved the submitted version.
