# Supplementary material for: Gene signature and prediction model of the mitophagy-associated immune microenvironment in renal ischemia-reperfusion injury
Source: Front Immunol. 2023 Mar 28;14:1117297. doi: 10.3389/fimmu.2023.1117297 (PMC10086170; doi:10.3389/fimmu.2023.1117297)
Supplement: Supplementary file 1 [file DataSheet_1.pdf]

FigureS1.

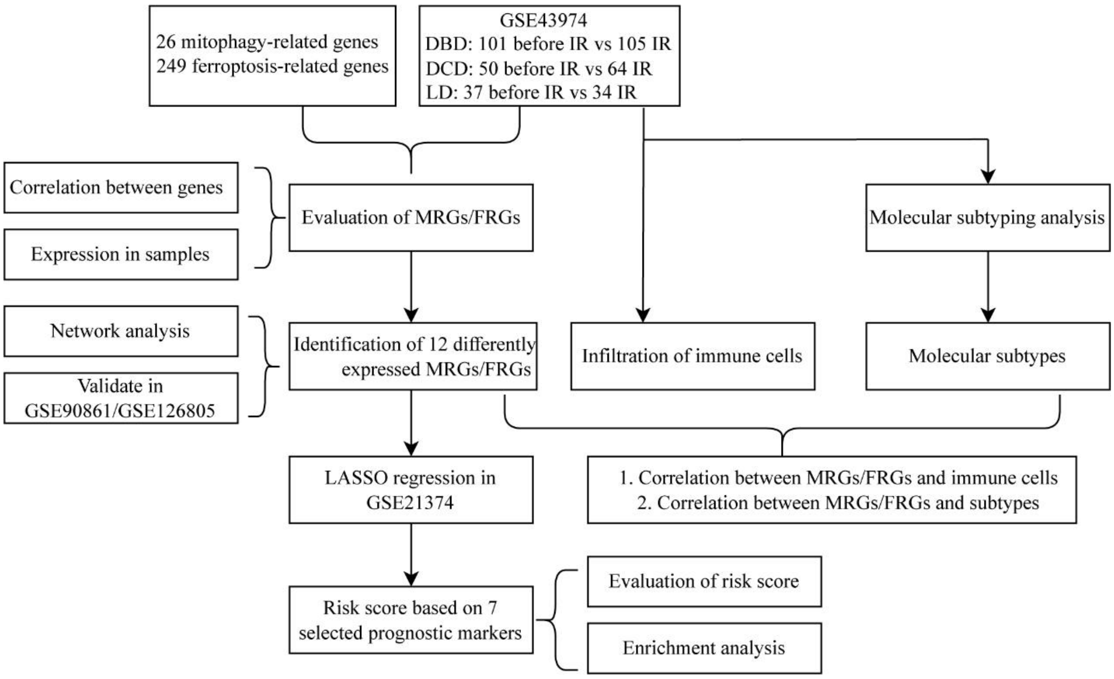

FigureS1. Flow chart.DBD, brain dead donors, brain dead population; DCD, cardiac dead donors, cardiac death population; LD, living donors, living donor kidney transplant population; IR, ischemia reperfusion, renal ischemia-reperfusion.

FigureS2.

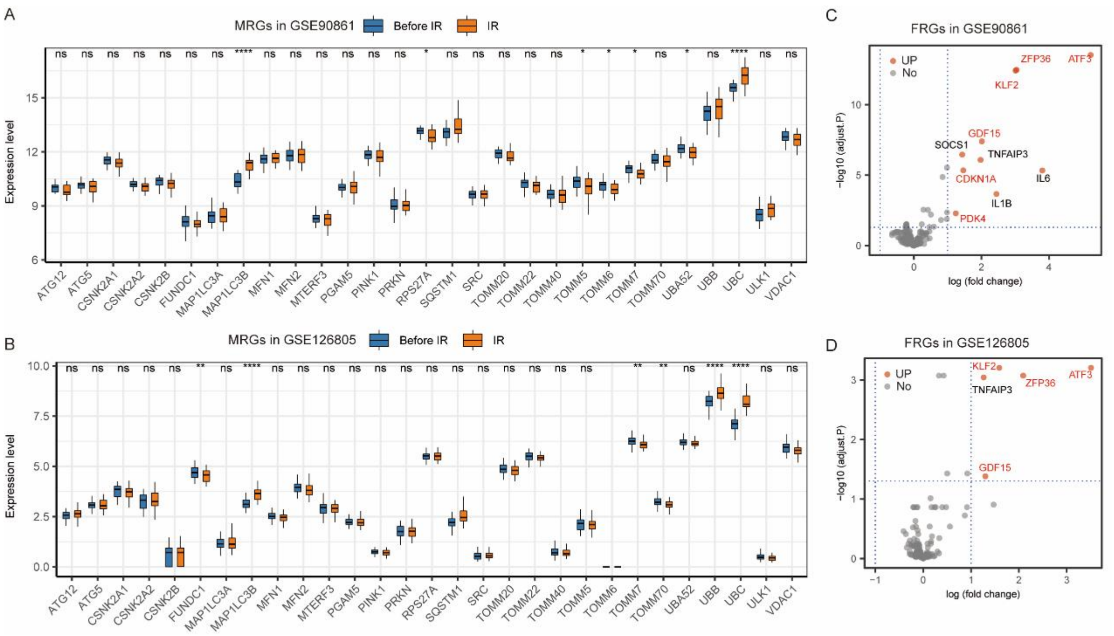

FigureS2. Validation of differentially expressed MRGs/FRGs. (A) Box plot of differentially expressed MRGs between IR and before IR samples in the GSE90861 dataset. (B) Box plot of differentially expressed MRGs between IR and before IR samples in the GSE126805 dataset. (C) Volcano plot of differentially expressed FRGs between IR and before IR samples in the GSE90861 dataset. (D) Volcano plot of differentially expressed FRGs between IR and before IR

samples in the GSE126805 dataset. Data were compared using the Wilcoxon test. *P < 0.05, **P < 0.01, ***P < 0.001, ****P < 0.0001. IR, ischemia reperfusion.

FigureS3.

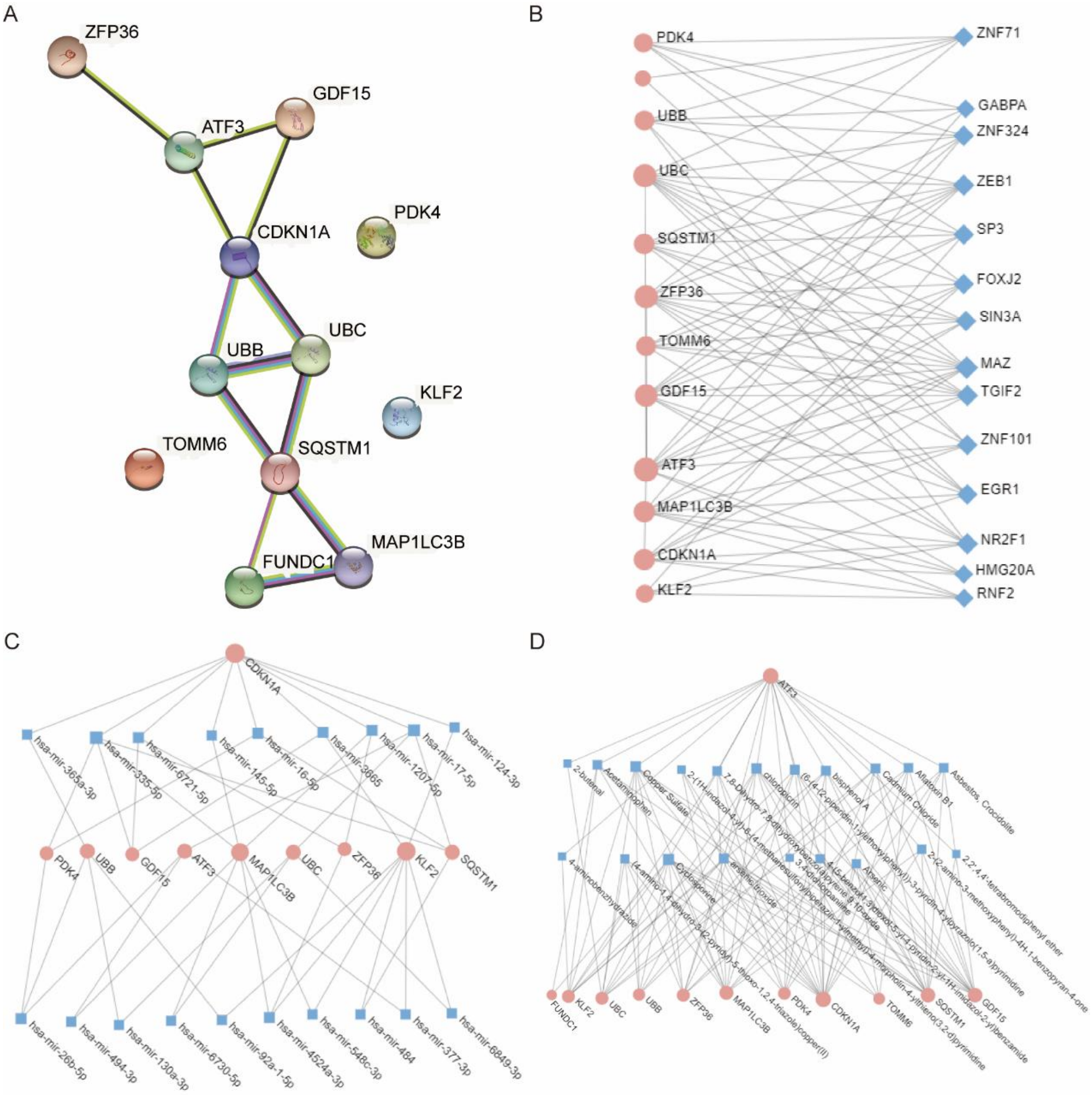

Figure S3. Interaction networks of differentially expressed MRGs/FRGs. (A) PPI network of differentially expressed MRGs/FRGs. (B) TF interaction network of differentially expressed MRGs/FRGs. Core network is shown after reducing the source network using the minimal network algorithm. Red indicates MRGs/FRGs, and blue indicates TF. (C) miRNA interaction network of differentially expressed MRGs/FRGs. Core network is shown after reducing the source network using the minimal network algorithm. Red indicates MRGs/FRGs, and blue indicates miRNAs. (D) Small molecule compound interaction network of differentially expressed MRGs/FRGs. Core network is shown after reducing the source network using the minimal network algorithm. Red indicates MRGs/FRGs, and blue indicates small molecule compounds.

FigureS4.

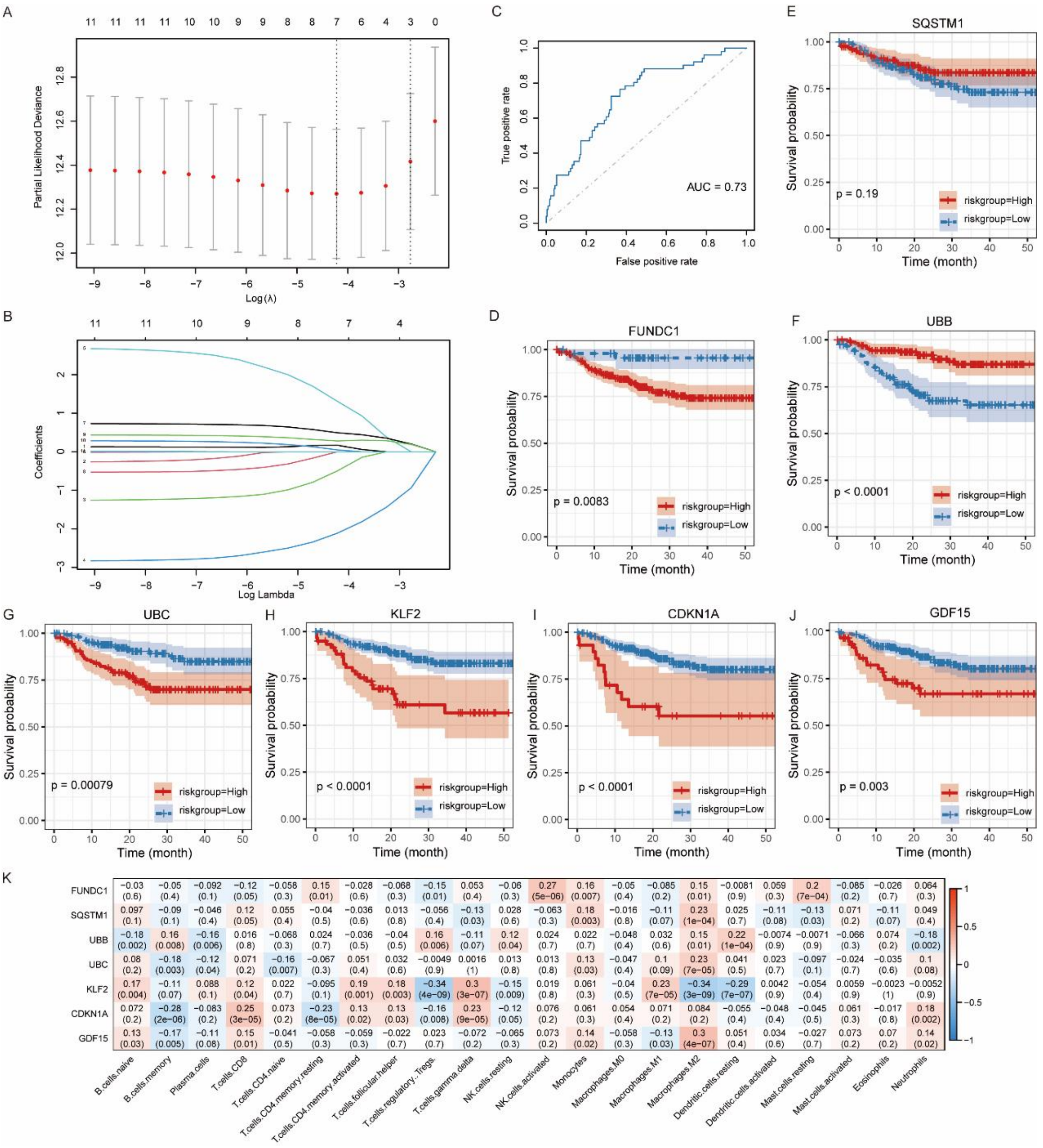

Figure S4. Prognostic marker selection and evaluation. (A-B) Prognostic markers were identified by LASSO regression, and the optimal λ was determined by partial likelihood bias of 10-fold cross-validation. (C) ROC analysis of the fitted values in predicting prognosis. (D-J) Survival curves (K-M method) of patients with low and high expression of the 7 prognostic markers (D) FUNDC1, (E) SQSTM1, (F) UBB, (G) UBC, (H) KLF2, (I) CDKN1A) and (J) GDF15. (K) Correlation matrices of the prognostic markers and extent of immune cell infiltration. Red indicates positive correlation and green indicates negative correlation. A darker color indicates higher correlation, and the correlation coefficients and P values are shown in the matrices. LASSO, least absolute shrinkage and selection operator; ROC, receiver operator characteristic curve.

Figure S5.

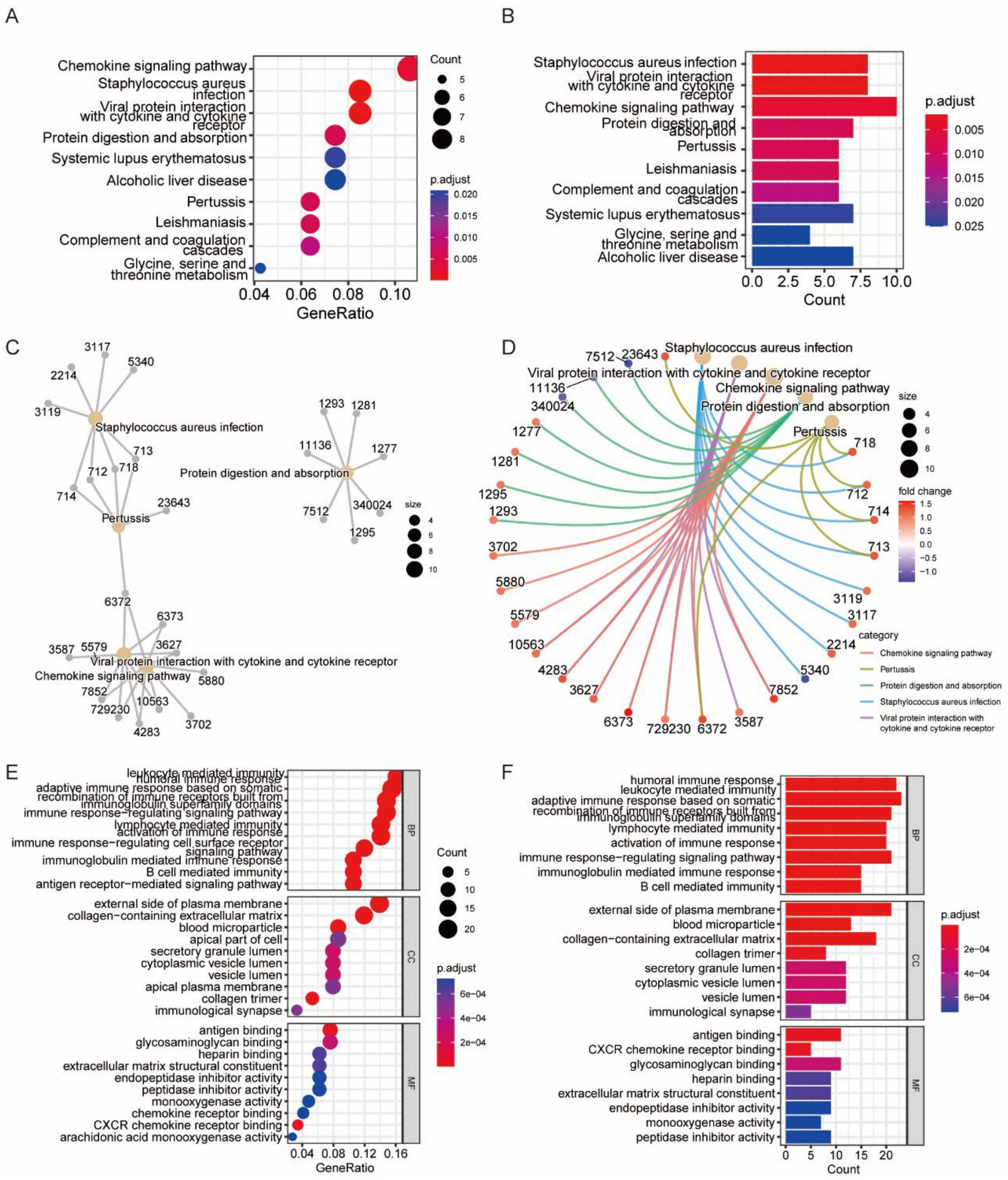

Figure S5. Enrichment analysis of differentially expressed genes between low and high RS groups. (A) Dot plot of KEGG results. A redder color indicates a smaller P value, and a larger dot indicates more genes enriched in a given pathway. (B) Bar chart of KEGG results. A redder color indicates a smaller P value, and the x-axis indicates the number of genes enriched in a given pathway. (C) Network chart of KEGG results. (D) Pie chart of KEGG results. (E) Dot plots of BP, CC and MF results of GO analysis. A redder color indicates a smaller P value, and a larger dot indicates more genes enriched in a given pathway. (F) Bar charts of BP, CC and MF results of GO analysis. A redder color indicates a smaller P value, and the x-axis indicates the number of genes enriched in a given pathway. (G) Network chart of GO results. KEGG, Kyoto Encyclopedia of Genes and Genomes; GO, gene ontology; BP, biological process; MF, molecular function; CC, cellular component.

FigureS6.

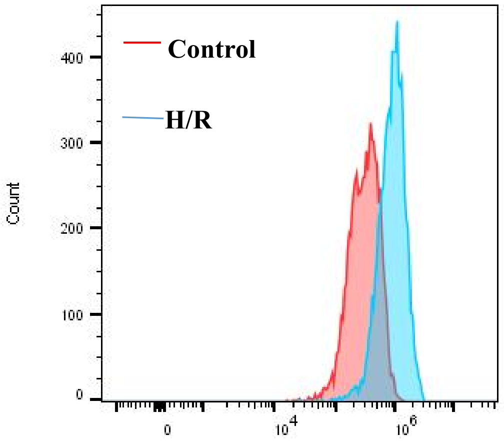

FigureS6 HK2 cells were analyzed for the intracellular ROS content by flow cytometry after 24 h of hypoxia and 4 h of reoxygenation.

FigureS7.

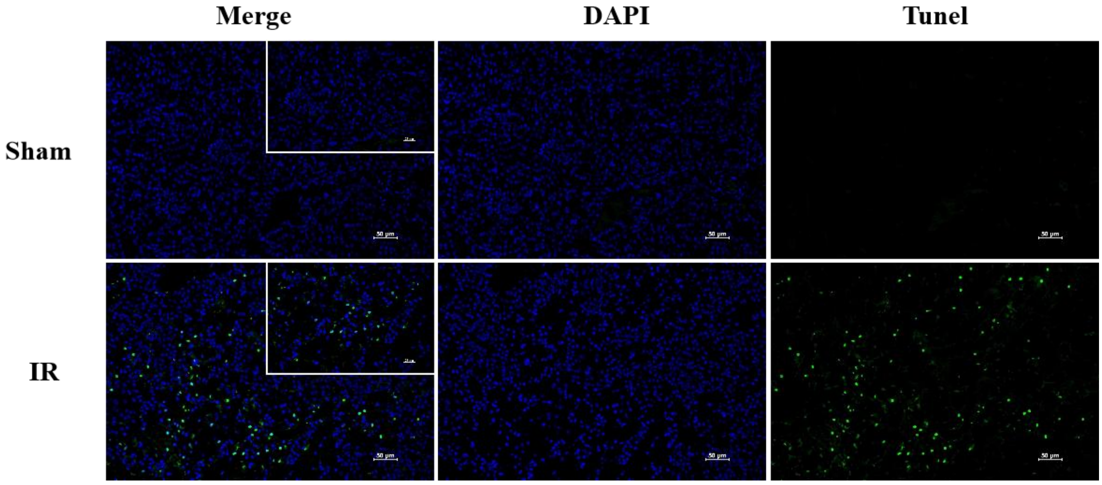

FigureS7.Cell apoptosis and necrosis were detected using Tunel staining in different groups.Scale bar, 50 um.

FigureS8.

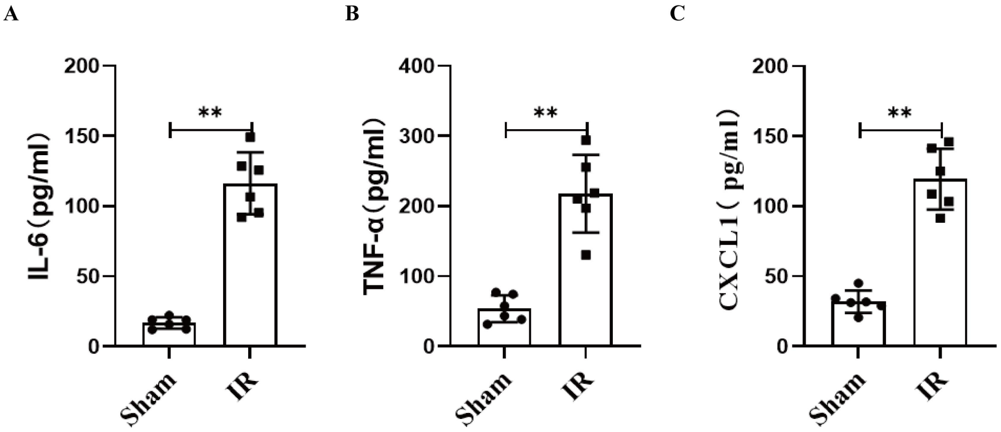

FigureS8.We tested the peripheral blood inflammatory factors in different groups of mice, including A, IL6, B, TNFa, C, and CXCL1.n = 6, n.s. not significant,*P<0.05,**P < 0.01 and***P < 0.001 between groups as indicated.

FigureS9.

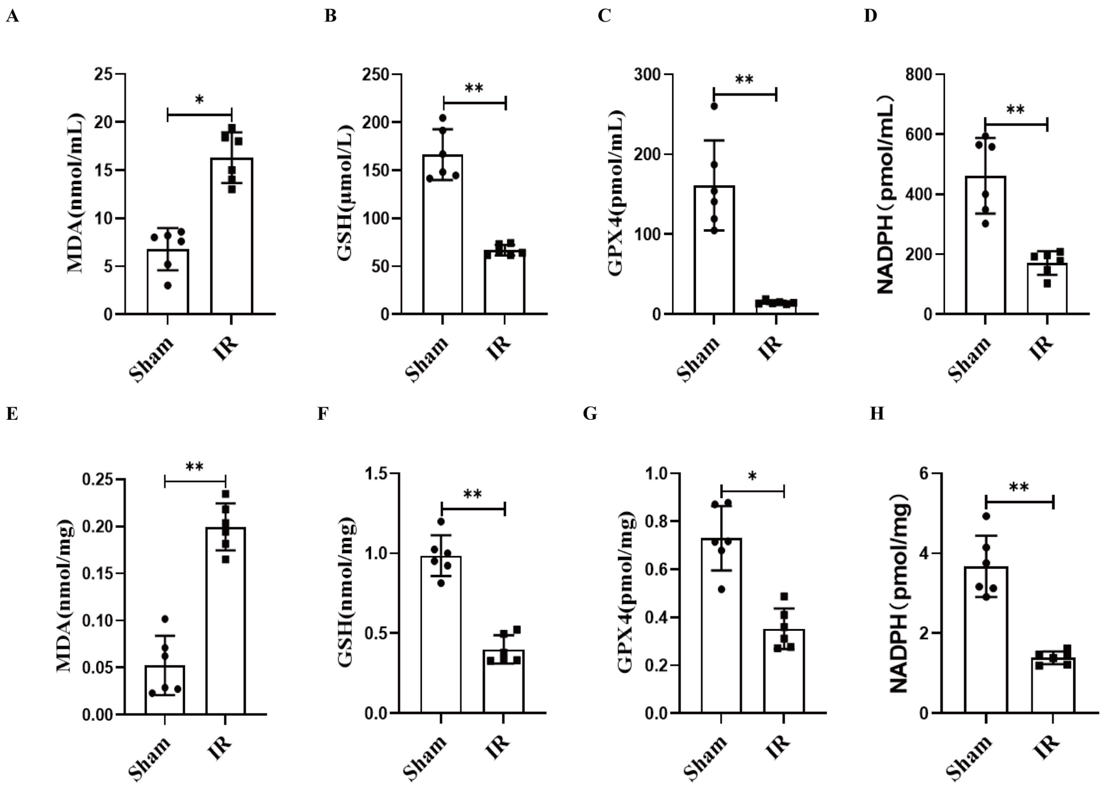

FigureS9.The levels of ferroptosis markers were detected in different groups.A,MDA in blood,B,GSH in blood,C,GPX4 in blood,D,NADPH in blood.E,MDA in kidney tissue
F,GSH in kidney tissue,G,GPX4 in kidney tissue,H,NADPH in kidney tissue
n = 6, n.s. not significant,*P<0.05,**P < 0.01 and***P < 0.001 between groups as indicated.

TableS1. List of mitophagy-related genes

Genes	Full-names
ATG12	Autophagy Related 12
ATG5	Autophagy Related 5
CSNK2A1	Casein Kinase 2 Alpha 1
CSNK2A2	Casein Kinase 2 Alpha 2
CSNK2B	Casein Kinase 2 Beta
FUNDC1	FUN14 Domain Containing 1
MAP1LC3A	Microtubule Associated Protein 1 Light Chain 3 Alpha

MFN1	Mitofusin 1
PGAM5	PGAM Family Member 5
PINK1	PTEN Induced Kinase 1
SQSTM1	Sequestosome 1
SRC	SRC Proto-Oncogene
TOMM20	Translocase Of Outer Mitochondrial Membrane 20
TOMM22	Translocase Of Outer Mitochondrial Membrane 22
TOMM5	Translocase Of Outer Mitochondrial Membrane 5
TOMM6	Translocase Of Outer Mitochondrial Membrane 6
TOMM7	Translocase Of Outer Mitochondrial Membrane 7
UBA52	Ubiquitin A-52 Residue Ribosomal Protein Fusion Product 1
ULK1	Unc-51 Like Autophagy Activating Kinase 1
MAP1LC3B	Microtubule Associated Protein 1 Light Chain 3 Beta
MFN2	Mitofusin 2
RPS27A	Ribosomal Protein S27a
TOMM40	Translocase Of Outer Mitochondrial Membrane 40
UBB	Ubiquitin B
UBC	Ubiquitin C
VDAC1	Voltage Dependent Anion Channel 1

TableS2. List of ferroptosis-related genes(FRGs)

Type	Gene name						
Driver	ABCC1	BAP1	ELOVL5	KDM6B	MTDH	RPL8	TSC1
	ACO1	BECN1	FADS1	KEAP1	MYB	SAT1	VDAC2
	ACSF2	BRD7	FADS2	KLF2	MYCN	SIRT1	WWTR1
	ACSL1	CCDC6	FAR1	LIFR	NCOA4	SIRT3	YAP1
	ACSL4	CD82	FBXW7	LONP1	NOX4	SLC11A2	YTHDC2
	ACVR1B	CDKN2A	G6PD	MAPK1	PANX1	SLC1A5	YY1AP1
	ADAM23	CDO1	GJA1	MAPK3	PAQR3	SLC25A28	ZEB1
	AGPS	CHAC1	GOT1	MAPK8	PEBP1	SLC39A7	
	ALOX12	CIRBP	GSK3B	MDM2	PEX10	SLC7A11	
	ALOX12B	CPEB1	GSTZ1	MDM4	PEX12	SMPD1	
	ALOX15	CS	H19	METTL14	PEX3	SNCA	
	ALOX15	CYB5R1	HDDC3	MIB1	PGD	SOCS1	

	B						
	ALOX5	CYGB	HMGB1	MIOX	PHKG2	TF	
	ALOXE3	DDR2	HMOX1	MIR15A	POR	TFRC	
	ANO6	DLD	IDH1	MIR214	PRKAA1	TGFBR1	
	ATF3	DNAJB6	IFNG	MIR302A	PRKAA2	TLR4	
	ATF4	DPP4	IL1B	MIR324	PRKCA	TNFAIP3	
	ATG5	EGFR	IL6	MIR375	PTPN6	TP53	
	ATG7	EGLN2	IREB2	MIR539	PVT1	TRIM26	
	ATM	ELAVL1	KDM5A	MIR761	QSOX1	TRIM46	
Suppressor	ABCC5	CAMKK2	DHODH	GLRX5	MEF2C	SIRT6	ZFP36
	ABHD12	CAV1	ECH1	GPX4	MEG8	SLC16A1	RNF113A
	ACSL3	CBS	ETV4	GSTM1	MGST1	SLC40A1	
	AIFM2	CD44	EZH2	HELLS	MIR130B	SOX2	
	AKR1C1	CDC25A	FABP4	HIF1A	MIR137	SQSTM1	
	AKR1C2	CDH1	FH	HSF1	MIR27A	SRC	
	AKR1C3	CDKN1A	FTH1	HSPA5	MIR424	SREBF1	
	AR	CHMP5	FTL	HSPB1	MIR545	SREBF2	
	ARF6	CHMP6	FTMT	IDH2	MIR670	STAT3	
	ARNTL	CISD1	FXN	ISCU	MIR9-1	STK11	
	ATF2	CISD2	FZD7	KDM3B	MIR9-3	SUV39H1	
	BCAT2	CISD3	GALNT14	KIF20A	MT1G	TFAM	
	BEX1	COPZ1	GCH1	KLHDC3	MTF1	TFAP2A	
	BRD4	CP	GCLC	LAMP2	MUC1	TMBIM4	
	CA9	DECR1	GDF15	LCN2	NEDD4	TP63	
	NEDD4L	NUPR1	PDSS2	PRDX6	RBMS1	TRIB2	
	NF2	OTUB1	PIR	PROM2	RRM2	TYRO3	
	NFE2L2	PANX2	PLA2G6	PTPN18	SCD	USP11	
	NFS1	PARK7	PML	RARRES2	SESN2	USP35	
	NQO1	PDK4	PPARA	RB1	SIAH2	VCP	

TableS3. Results of the KEGG analysis of differentially expressed genes by risk score grouping

ID	Description	GeneRatio	BgRatio	p.adjust	Count
hsa05150	Staphylococcus aureus infection	8/94	96/8165	0.0018566	8

hsa04061	Viral protein interaction with cytokine and cytokine receptor	8/94	100/8165	0.0018566	8
hsa04062	Chemokine signaling pathway	10/94	192/8165	0.0046151	10
hsa04974	Protein digestion and absorption	7/94	103/8165	0.0084442	7
hsa05133	Pertussis	6/94	76/8165	0.0084442	6
hsa05140	Leishmaniasis	6/94	77/8165	0.0084442	6
hsa04610	Complement and coagulation cascades	6/94	86/8165	0.0132103	6
hsa05322	Systemic lupus erythematosus	7/94	136/8165	0.0243081	7
hsa00260	Glycine, serine and threonine metabolism	4/94	40/8165	0.0250487	4
hsa04936	Alcoholic liver disease	7/94	142/8165	0.0250487	7

Table S4. Results of GO analysis of differentially expressed genes in risk score grouping

ONTOLOGY	ID	Description	GeneRatio	BgRatio	p.adjust	Count
BP	GO:0006959	humoral immune response	22/142	317/18800	6.14E-12	22
BP	GO:0002443	leukocyte mediated immunity	23/142	457/18800	4.73E-10	23
BP	GO:0002460	adaptive immune response based on somatic recombination of immune receptors built from immunoglobulin superfamily domains	21/142	370/18800	4.73E-10	21
BP	GO:0002449	lymphocyte mediated immunity	20/142	365/18800	2.51E-09	20
BP	GO:0002253	activation of immune response	20/142	386/18800	5.54E-09	20
BP	GO:0002764	immune response-regulating signaling pathway	21/142	482/18800	2.98E-08	21
BP	GO:0016064	immunoglobulin mediated immune response	15/142	216/18800	2.98E-08	15
BP	GO:0019724	B cell mediated immunity	15/142	219/18800	3.17E-08	15
BP	GO:0002768	immune response-regulating cell	17/142	328/18800	1.15E-07	17

		surface receptor signaling pathway				
BP	GO:0050851	antigen receptor-mediated signaling pathway	15/142	244/18800	1.15E-07	15
CC	GO:0009897	external side of plasma membrane	21/151	455/19594	1.04E-08	21
CC	GO:0072562	blood microparticle	13/151	147/19594	1.21E-08	13
CC	GO:0062023	collagen-containing extracellular matrix	18/151	429/19594	4.14E-07	18
CC	GO:0005581	collagen trimer	8/151	86/19594	1.74E-05	8
CC	GO:0034774	secretory granule lumen	12/151	322/19594	0.0002715	12
CC	GO:0060205	cytoplasmic vesicle lumen	12/151	325/19594	0.0002715	12
CC	GO:0031983	vesicle lumen	12/151	327/19594	0.0002715	12
CC	GO:0001772	immunological synapse	5/151	44/19594	0.0005198	5
CC	GO:0016324	apical plasma membrane	12/151	358/19594	0.0005198	12
CC	GO:0045177	apical part of cell	13/151	424/19594	0.0005382	13
MF	GO:0003823	antigen binding	11/145	174/18410	3.99E-05	11
MF	GO:0045236	CXCR chemokine receptor binding	5/145	18/18410	3.99E-05	5
MF	GO:0005539	glycosaminoglycan binding	11/145	234/18410	0.0002996	11
MF	GO:0008201	heparin binding	9/145	168/18410	0.0006435	9
MF	GO:0005201	extracellular matrix structural constituent	9/145	172/18410	0.0006435	9
MF	GO:0004866	endopeptidase inhibitor activity	9/145	180/18410	0.0007241	9
MF	GO:0004497	monooxygenase activity	7/145	103/18410	0.0007241	7
MF	GO:0030414	peptidase inhibitor activity	9/145	187/18410	0.0007241	9
MF	GO:0008391	arachidonic acid monooxygenase activity	4/145	21/18410	0.0007241	4
MF	GO:0042379	chemokine receptor binding	6/145	71/18410	0.0007241	6

Table S5. qPCR primer

Gene Name	F primer	R primer

FUNDC1	CTGGCTGGTGTGCAGGATT	TGAAGAAGAAGGAAACCACCACC
SQSTM1	AGTAACACTCAGCCAAGCAGC	CGACTCCATCTGTTCCTCTGG
UBB	CGAGAACGTGAAGGCCAAGA	GCAGGGTTGACTCCTTCTGG
UBC	ACCAAGAAGGTCAAACAGGAAGA	CACACCCAAGAACAAGCACAA
KLF2	GAGCCTATCTTGCCGTCCTT	TGTTTAGGTCCTCATCCGTGC
CDKN1A	TTCCGCACAGGAGCAAAGTG	ACGAAGTCAAAGTTCCACCGT
GDF15	AGCTGGAACTGCGCTTACG	TCAAGAGTTGCCTGCACAGT

TableS6. Database website

Name	website
Pathway Unification	https://pathcards.genecards.org/
FerrDb	http://www.zhounan.org/ferrdb/current/operations/download.html
STRING	http://www.string-db.org/
NetworkAnalyst	https://www.networkanalyst.ca/
TF databases ENCODE	http://cistrome.org/BETA/
miRTarBase v8.0	https://mirtarbase.cuhk.edu.cn
Comparative Toxicogenomics Database	http://ctdbase.org/
CIBERSORTx	https://cibersortx.stanford.edu/

Methods Supplementary Material

The microarray platforms used were GPL10558 Illumina HumanHT-12 V4.0 expression beadchip for GSE43974, GPL18573 Illumina NextSeq 500 (Homo sapiens) for GSE90861, GPL21290 Illumina HiSeq 3000 (Homo sapiens) for GSE126805, and GPL570 [HG-U133_Plus_2] Affymetrix Human Genome U133 Plus 2.0 Array for GSE21374.

DEGs were uploaded to NetworkAnalyst (https://www.networkanalyst.ca/) and analyzed by the TF-gene interactions, Gene-miRNA interactions, and Protein-chemical interaction modules using TF databases ENCODE (http://cistrome.org/BETA/), miRTarBase v8.0 (https://mirtarbase.cuhk.edu.cn) and Comparative Toxicogenomics Database (http://ctdbase.org/) .

A list of mitophagy-related genes (MRGs) was acquired from the Pathway Unification database (https://pathcards.genecards.org/), and a final total of 26 MRGs were extracted from the transcriptomic data of GSE43974. A list of ferroptosis-related genes(FRGs) was acquired from FerrDb (http://www.zhounan.org/ferrdb/current/operations/download.html)

Non-negative matrix factorization (NMF) is a group of algorithms that factorize a given non-negative matrix V into two non-negative matrices W and H such that V=W*H. In matrix V, each column represents an observation and each row represents a feature. Matrix W is the basis matrix and matrix H is the coefficient or weighted matrix. Substitution of the original matrix with the coefficient matrix H reduces the dimension of the original matrix and data features.

Least absolute shrinkage and selection operator (LASSO) is a shrinkage estimation method. LASSO provides a more refined model by constructing a penalty function and shrinking some coefficient to zero. This method retains subset shrinkage and is a biased estimate for complex collinear data.

The risk score (RS) of each case was calculated by:

$$\mathrm{RS} = \sum_{\mathrm{i}=1}^{\mathrm{n}} \mathrm{Coef_i} \times \mathrm{Exp_i}$$

where Coef is the LASSO regression coefficient and Exp is the RNA expression level (log2 transformed).

Kyoto Encyclopedia of Genes and Genomes (KEGG) is a commonly used database that contains information on genomes, biological pathways, diseases and drugs. Gene Ontology (GO) is a functional annotation method commonly used in large-scale functional enrichment studies that cover biological process (BP), molecular function (MF), and cellular component (CC).
